# The association between physical activity and cognitive function in the elderly in rural areas of northern China

**DOI:** 10.3389/fnagi.2023.1168892

**Published:** 2023-06-20

**Authors:** Xueyan Wang, Jiesong Zhang, Chen Chen, Zhonghai Lu, Dongfeng Zhang, Suyun Li

**Affiliations:** Department of Epidemiology and Health Statistics, School of Public Health, Qingdao University, Qingdao, Shandong, China

**Keywords:** physical activity, cognitive function, threshold effect, saturation effect, elderly people

## Abstract

**Background:**

Physical activity plays an important role in cognitive function in older adults, and the threshold effect and saturation effect between physical activity and cognitive function are unclear.

**Objective:**

The purpose of this study was to explore the threshold effect and saturation effect between physical activity and cognitive function in the elderly.

**Methods:**

The International Physical Activity Questionnaire (IPAQ) was used to measure moderate-intensity physical activity and vigorous-intensity physical activity and total physical activity in older adults. Cognitive function assessment uses the Beijing version of the Montreal Cognitive Assessment Scale (MoCA). The scale consists of seven parts: visual space, naming, attention, language, abstract ability, delayed recall and orientation, for a total of 30 points. The total score of the study participants < 26 was defined as the optimum cutoff point for a definition of mild cognitive impairment (MCI). The multivariable linear regression model was used to initially explore the relationship between physical activity and total cognitive function scores. The logistic regression model was used to assess the relationship between physical activity and cognitive function dimensions and MCI. The threshold effect and saturation effect between the total physical activity and the total cognitive function scores were investigated by smoothed curve fitting.

**Results:**

This cross-sectional survey had a total of 647 participants aged 60 years and older (mean age: 73 years, female: 53.7%). Participants’ higher level of physical activity were associated with higher visual space, attention, language, abstract ability, and delayed recall scores (*P* < 0.05). Physical activity was not statistically associated with naming and orientation. Physical activity was a protective factor for MCI (*P* < 0.05). Physical activity was positively correlated with total cognitive function scores. There was a saturation effect between total physical activity and total cognitive function scores, and the saturation point was 6546 MET × min/wk.

**Conclusion:**

This study showed a saturation effect between physical activity and cognitive function, and determined an optimal level of physical activity to protect cognitive function. This finding will help update physical activity guidelines based on cognitive function in the elderly.

## Introduction

With the increasement of life expectancy in global population and the decline of the fertility rates, the proportion of people aged 60 years old or over is expected to double between 2013 (11.7%) and 2050 (21.1%) according to a United Nations report (United Nations, 2013). Age-related cognitive decline caused by aging even could progress to pathological mild cognitive impairment (MCI) and dementia in severe cases. MCI is known as the negative and pathological cognitive changes that exceed expectations during normal aging, which is a transitional state between normal aging and dementia (American Psychiatric Association, 1994). A cross-sectional study has shown that the overall population with MCI and dementia is estimated to account for more than one-fifth of adults aged 60 years or older in China (Jia et al., 2020). Besides, previous study has revealed a higher prevalence rate of dementia in older adults living in rural areas rather than urban dwellers in China (Jia et al., 2020). There exists no effective treatment for dementia, so interventions targeting MCI are necessary to prevent the onset of dementia in China, especially in rural areas.

The risk of cognitive decline can be reduced by management of modifiable risk factors (Barnes and Yaffe, 2011; Ngandu et al., 2015). Previous studies have shown that many individual and environmental factors may be associated with cognitive function, such as age (World Health Organization [WHO], 2022a), education (Cagney and Lauderdale, 2002), smoking (Stewart et al., 2006), alcohol (Edelstein et al., 1998), physical activity (PA) (Zou et al., 2019), and depression (Ismail et al., 2017).

Physical activity, as modifiable lifestyle factors, can diminish or prevent cognitive decline associated with aging (Brisswalter et al., 2002; Colcombe and Kramer, 2003; Voss et al., 2013). However, due to the differences in methodology and classification of various researches, there also exist few evidence suggests that improved cognitive function is not always related to PA in older adults (Cott et al., 2002; Miu et al., 2008; Hoffmann et al., 2016). PA may improve cognitive function in several manner, such as by promoting neurogenesis, angiogenesis, synaptic plasticity, reducing pro-inflammatory processes, and reducing cellular damage caused by oxidative stress (Rasmussen et al., 2009). According to World Health Organization guidelines, adults 65 years of age and older should engage in at least 150 min of moderate-intensity physical activity (MPA) or at least 75 min of vigorous-intensity physical activity (VPA), or a combination of moderate-intensity and vigorous-intensity activities to achieve an equivalent amount of physical activity (MVPA) (600 metabolic equivalent minutes per week) should be performed each week (World Health Organization [WHO], 2022b). Although there are currently many studies exploring the relationship between PA and cognitive function, it is still uncertain whether there is a linear or non-linear relationship between the PA and cognitive function, as well as whether there are threshold effects and saturation effects for the association.

In addition, the relationship between PA and cognitive function could be modified by gender probably. For example, previous studies have found that PA has a greater impact on cognitive function ability in older women than in older men (Brisswalter et al., 2002), supporting the hypothesis that gender may be a potential moderator of relationship between PA and cognitive function (Bell et al., 2019). Further studies are needed to explore whether the association between PA and cognitive function was modified by gender on the elderly in rural areas of China.

To sum up, the aim of this study is to investigate the association between PA and cognitive function and to explore whether the association was modified by gender on the elderly in rural areas of northern China. In addition, we will further explore if the threshold effect and saturation effect exist in the relationship of PA and cognitive function and find the optimal PA level to protect cognitive function.

## Materials and methods

### Study subjects

In this cross-sectional study, older adults were used to select as participants from 17 villages in Jimo District, Qingdao City. A face-to-face interview format was used to interview the study participants using questionnaires to obtain information on various aspects of their cognitive functioning, demographic characteristics, and other environmental factors. Based on the simple random sampling method and inclusion exclusion criteria, 650 people participated in the preliminary survey. Among them, 3 participants did not complete all the questionnaires, and the response rate was 99.54%. In the end, a total of 647 participants participated in the study. Inclusion criteria: ➀ permanent Han Chinese residents of Qingdao (>5 years); ➁ aged ≥ 60 years; ➂ independent in daily life; ➃ agree and sign the written informed consent. Exclusion criteria: ➀ suffer from brain diseases such as stroke, brain tumor, epilepsy; ➁ suffer from serious physical and mental diseases; ➂ hypothyroidism; ➃ severe physical disability and visual and hearing impairment resulting in inability to cooperate; ➄ those who already suffer from dementia or AD; ➅ those who refuse to cooperate.

This study was in compliance with Helsinki declaration and approved by the Ethics Committee of Qingdao University Medical College.

### Cognitive function test

Cognitive function assessments were tested using the Beijing version of the Montreal Cognitive Assessment (MoCA) (Nasreddine et al., 2005). MoCA is an assessment tool developed by Nasreddine in 2004 for rapid screening of MCI. The scale consists of seven parts: visual space (0–5 points), naming (0–3 points), attention (0–6 points), language (0–3 points), abstract ability (0–2 points), delayed recall (0–5 points) and orientation (0–6 points), a total of 30 points and <26 points are defined as the optimum cutoff point for a definition of MCI. If the number of years of education ≤ 12 years, 1 point can be added. Based on this score criterion, participants were divided into case (MCI group, *n* = 411) group and control (normal cognitive function group, *n* = 236) group. The scale measures a wide range of respondents’ cognitive function across all dimensions, with higher scores indicating better cognitive function. The Cronbach α coefficient of MoCA Beijing is 0.74.

### Physical activity test

Physical activity was measured using the short version of International Physical Activity Questionnaire (IPAQ) (Craig et al., 2003). IPAQ assigned metabolic equivalent (MET) corresponding to different intensities of PA by asking the study subjects about the frequency of the week (d/wk) and the time of day (min/d) for that intensity. The MET was 3.3 for walking, 4.0 for MPA, and 8.0 for VPA. The PA level for each intensity and the total PA level were calculated separately according to the formula. The IPAQ formula is defined as follows.

➀ Walking MET × min/wk = 3.3 × Walking weekly frequency (d/wk) × Walking daily time (min/d)

➁ MPA MET × min/wk = 4.0 × MPA weekly frequency (d/wk) × MPA daily time (min/d)

➂ VPA MET × min/wk = 8.0 × VPA weekly frequency (d/wk) × VPA daily time (min/d)

➃ MVPA MET × min/wk = ➁ + ➂

➄ Total PA MET × min/wk = ➀ + ➁ + ➂

We divided the participants of this study into two groups according to the minimum amount of MVPA (600 MET × min/wk) of the WHO guidelines (Below PA Recommendation group: <600 MET × min/wk; Over PA Recommendation group: ≥600 MET × min/wk). IPAQ has a Cronbach α coefficient of 0.56.

### Covariates

Age, gender (male or female), education level (≤12 years or >12 years), occupation status (farmers or others), marital status (married or others), smoking status (yes or no), and alcohol drinking status (yes or no) were collected through questionnaires. Participants were classified as smokers if they smoked at least 100 cigarettes in their lifetime, and those who smoked < 100 cigarettes in their lifetime were classified as non-smokers (Gu et al., 2020). Participants were classified as alcohol drinkers if they drank at least 1 time per week, and those who never drank or less than 1 drink per week were classified as non-alcohol drinkers (Sun et al., 2019). Diabetes, hypertension, and depression were defined based on self-reported physician diagnoses and were defined as yes or no.

### Statistical analysis

In this study, the general characteristics of the study subjects were described in the form of frequency and composition ratios for the qualitative variable, while for the quantitative variable, the mean ± SD was used. Student’s *t*-test or Wilcoxon rank-sum test was used to compare the mean levels of continuous variables between below PA recommendation group and over PA recommendation group based on their distribution. Pearson chi-squared test or Fisher’s exact test was performed to compare the distribution of the categorical variables between below PA recommendation group and over PA recommendation group.

The multivariable linear regression model was used to evaluate the association between PA group and total cognitive function scores. Binary logistic regression model was used to analyze the association between PA group and MCI. Ordered logistic regression model was used to analyze the association between PA group and cognitive function score of different dimensions.

The smooth curve was used to fit the correlation between total PA and total cognitive function scores. If there is a non-linear association, the inflection point is determined using the recursive method according to the principle of maximum likelihood method. Confidence intervals for the thresholds were determined by using the Bootstrap resampling method. Then, a segmented multivariable linear regression model was built on both edges of the inflection point, and a log-likelihood ratio test was used to verify the multivariable linear regression model and the two-piecewise multivariable linear model. The threshold effect means that the relationship between X and Y only manifests itself when X accumulates to a certain amount. The saturation effect means that after a certain point, the effect of X on Y no longer increases with the increase of X (Chen, 2016). Covariates in the models include age, gender, education, marital status, occupation status, smoking status, alcohol drinking status, depression, hypertension and diabetes. The variance inflation factor (VIF) was used to evaluate the multicollinearity of the independent variables.

To determine whether gender factors alter the relationship between total PA and MCI, we performed a stratified analysis of different gender groups. A binary logistic regression was used to assess the effect size of the risk of MCI in each group after stratification. By adding interaction terms to the logistic regression model, the interaction effect of total PA and gender on MCI was estimated.

All data were analyzed using Stata, version 15.0, EmpowerStats, version 4.1, and R, version 3.5.1. Hypothesis testing was performed using a two-sided test, with *P* < 0.05 as the criterion for statistical significance.

## Results

### Sample characteristics

The demographic characteristics and different PA level of 647 participants were described in Table 1. 78.8% of the participants in this survey could meet the minimum recommended PA amount by WHO. Compared with the Below PA Recommendation group, the Over PA Recommendation group had significant differences in age, education, marital status and depression. Participants in the high-amount PA group were likely to be younger, less educated, married, and have a lower prevalence of depression than the low-amount PA group (*P* < 0.05). Distribution of gender, occupation, smoking status, alcohol drinking status, hypertension and diabetes between the two groups was not significantly different.

**TABLE 1:** The characteristics of study population.

Characteri-stics	Participants	Below PA recommen -dation group	Over PA recommen -dation group	*P*
Participants	647	137 (21.2)	510 (78.8)	
Age, mean (SD)^a^	73 ± 6.1	75 ± 6.6	72 ± 5.7	0.001
Gender, *n* (%)^b^				0.800
Male	299	62 (45.3)	237 (46.5)	
Female	348	75 (54.7)	273 (53.5)	
Education, *n* (%)^b^				0.046
≤12 years	616	126 (91.9)	490 (96.1)	
>12 years	31	11 (8.1)	20 (3.9)	
Occupation, *n* (%)^b^				0.344
Farmers	505	111 (81.1)	394 (77.3)	
Others	142	26 (18.9)	116 (22.7)	
Marital status, *n* (%)^b^				0.011
Married	501	95 (69.3)	406 (79.6)	
Others	146	42 (30.7)	104 (20.4)	
Smoking status, *n* (%)^b^				0.559
Yes	207	41 (29.9)	166 (32.5)	
No	440	96 (70.1)	344 (67.5)	
Alcohol drinking status, *n* (%)^b^				0.239
Yes	152	27 (19.7)	125 (24.5)	
No	495	110 (80.3)	138 (75.5)	
Depression, *n* (%)^b^				0.001
Yes	189	62 (45.3)	127 (24.9)	
No	458	75 (54.7)	383 (75.1)	
Hypertension, *n* (%)^b^				0.060
Yes	294	72 (52.6)	222 (43.5)	
No	353	65 (47.4)	288 (56.5)	
Diabetes, *n* (%)^b^				0.321
Yes	72	12 (8.8)	60 (11.8)	
No	575	125 (91.2)	450 (88.2)	

PA, physical activity; “Below PA recommendation” in the table represents “the PA amount performed by subjects is less than the WHO recommended PA amount”; “Over PA recommendation” in the table represents “the PA amount performed by subjects is more than the WHO recommended PA amount.”

^a^*P* value was tested by Wilcoxon rank sum test or Student’s *t*-test;

^b^*P* value was tested by Chi-square test or Fisher’s exact.

### Analysis of PA group and cognitive function

**FIGURE 1:**
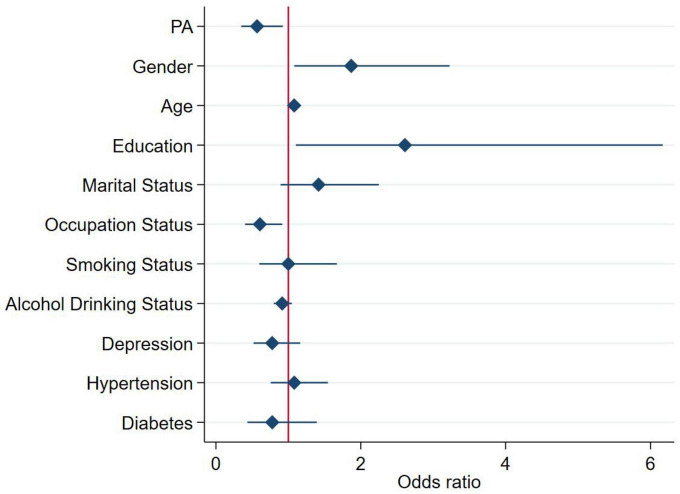
Association between physical activity (PA) group and MCI. For the analysis, we adjusted for age, gender (male or female), education level (≤12 years or >12 years), occupation status (farmers or others), marital status (married or others), smoking status (yes or no), alcohol drinking status (yes or no), depression, hypertension, and diabetes. The square and solid lines represent the estimated OR and its 95% confidence interval, respectively.

**FIGURE 2:**
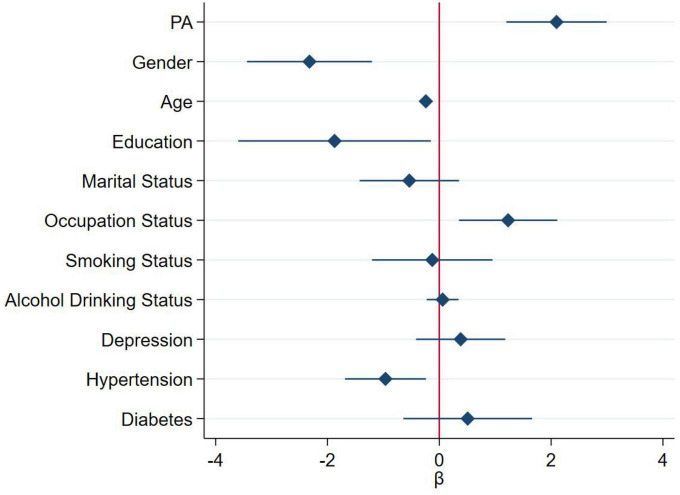
Association between physical activity (PA) group and total cognitive function scores (continuous variable). For the analysis, we adjusted for age, gender (male or female), education level (≤12 years or >12 years), occupation status (farmers or others), marital status (married or others), smoking status (yes or no), alcohol drinking status (yes or no), depression, hypertension, and diabetes. The diamond and solid lines represent the estimated ß and its 95% confidence interval, respectively.

The results of the association between PA group and cognitive function score of different dimensions and MCI were shown in Table 2 and Figure 1. Table 3 and Figure 2 showed the association between PA group and total cognitive function scores. The univariate logistic regression showed that PA was a risk factor for visual space, attention, language, abstract ability, delayed recall and orientation (*P* < 0.05). This suggests that higher level of PA was associated with better cognitive function. After adjustment for confounding factors, visual space, attention, language, abstract ability and delayed recall were still positively correlated with PA. Regardless of univariate or multivariable logistic regression analysis, the PA is a protective factor for MCI, indicates individuals with higher level of PA were at lower risk of developing MCI (*P* < 0.05). Multivariable linear regression analysis showed that PA was positively correlated with the total cognitive function scores before and after covariate adjustment, indicating that higher PA level was associated with higher total cognitive function scores, and better cognitive function (*P* < 0.05).

**TABLE 2:** Association between PA group and cognitive function score of different dimensions and MCI.

MoCA test	OR (95%CI)^a^	*P* ^a^	OR (95%CI)^b^	*P* ^b^	OR (95%CI)^c^	*P* ^c^
Visual space^d^	1.92 (1.36–2.72)	0.001	1.56 (1.09–2.20)	0.013	1.77 (1.23–2.54)	0.002
Naming^d^	1.68 (0.85–3.31)	0.135	1.48 (0.73–3.02)	0.277	1.44 (0.69–2.99)	0.327
Attention^d^	1.90 (1.34–2.69)	0.001	1.59 (1.11–2.29)	0.012	1.66 (1.14–2.44)	0.009
Language^d^	2.82 (1.87–4.24)	0.001	2.31 (1.51–3.53)	0.001	2.27 (1.44–3.53)	0.001
Abstract ability^d^	1.89 (1.33–2.69)	0.001	1.60 (1.11–2.29)	0.011	1.76 (1.21–2.56)	0.003
Delayed recall^d^	1.81 (1.28–2.55)	0.001	1.56 (1.10–2.21)	0.012	1.64 (1.14–2.36)	0.007
Orientation^d^	1.80 (1.26–2.56)	0.001	1.46 (1.01–2.09)	0.042	1.34 (0.92–1.95)	0.126
MCI^e^	0.48 (0.31–0.74)	0.001	0.56 (0.36–0.89)	0.015	0.56 (0.34–0.91)	0.019

PA, physical activity; PA group, below PA recommendation group, and over PA recommendation group; MCI, mild cognitive impairment.

^a^Crude results.

^b^Adjusted for age and gender (male or female).

^c^Adjusted for age, gender (male or female), education level (≤12 years or >12 years), occupation status (farmers or others), marital status (married or others), smoking status (yes or no), alcohol drinking status (yes or no), depression, hypertension, and diabetes.

^d^*P* value was tested by ordered logistic regression; ^e^*P* value was tested by binary logistic regression.

**TABLE 3:** Association between PA group and total cognitive function scores.

PA group	β (95%CI)^a^	*P* ^a^	β (95%CI)^b^	*P* ^b^	β (95%CI)^c^	*P* ^c^
Below PA recommendation group	Reference		Reference		Reference	
Over PA recommendation group	2.85 (1.90–3.79)	0.001	2.03 (1.15–2.91)	0.001	2.11 (1.21–3.00)	0.001

PA, physical activity; PA group, below PA recommendation group and over PA recommendation group.

^a^Crude results.

^b^Adjusted for age and gender (male or female).

^c^Adjusted for age, gender (male or female), education level (≤12 years or >12 years), occupation status (farmers or others), marital status (married or others), smoking status (yes or no), alcohol drinking status (yes or no), depression, hypertension, and diabetes.

### Threshold effect and saturation effect analysis

**FIGURE 3:**
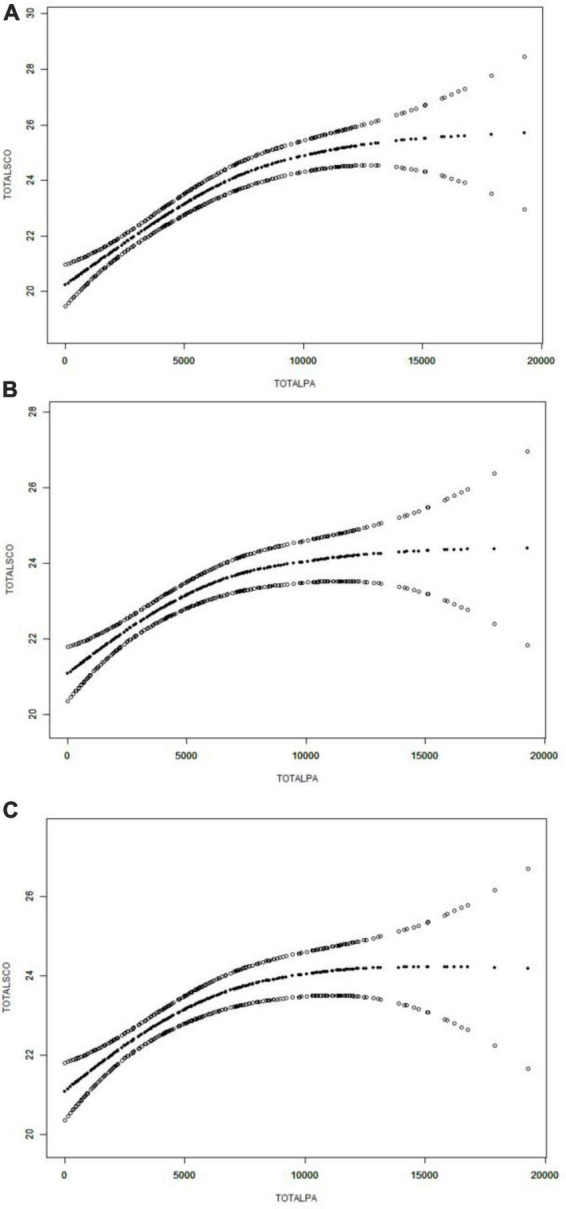
Association of total physical activity (PA) with total cognitive function scores. **(A)** Crude model did not adjust any confounders. **(B)** Adjusted for age and gender (male or female). **(C)** Adjusted for age, gender (male or female), education level (≤12 years or >12 years), occupation status (farmers or others), marital status (married or others), smoking status (yes or no), alcohol drinking status (yes or no), depression, hypertension, and diabetes. Smooth curve fitting was used to estimate the associations. The middle line indicates the estimated value, and the upper and lower lines indicate the corresponding 95% confidence intervals.

The total cognitive function scores had a non-linear association with total PA, with obvious saturation effect and saturation point (Figure 3 and Table 4). When the total PA was 6546 MET × min/wk, the smooth curve of the total cognitive function scores peaked. When the total PA was 6546 MET × min/wk, the total cognitive function scores increased by 0.0005 points per 1-MET × min/wk increase in PA (β = 0.0005; 95% CI = 0.0003 to 0.0007; *P* < 0.001). When total PA exceeds the saturation point, the total cognitive function scores showed a non-significant escalating trend (β = −0.0000; 95% CI = −0.0002 to 0.0002; *P* = 0.9192).

**TABLE 4:** Threshold effect and saturation effect analysis of total PA on total cognitive function scores.

Total PA MET × min/wk	Total cognitive function scores	
	**β (95%CI)**	***P***
Standard multivariable linear regression model	0.0002 (0.0001, 0.0003)	<0.001
Inflection point (k)	6546	
Less than k*	0.0005 (0.0003, 0.0007)	<0.001
More than k	−0.0000 (−0.0002, 0.0002)	0.9192
*P* for log likelihood ratio test		0.003

Model is adjusted for age, gender (male or female), education level (≤12 years or >12 years), occupation status (farmers or others), marital status (married or others), smoking status (yes or no), alcohol drinking status (yes or no), depression, hypertension and diabetes.

*Fitting model by two-piecewise multivariable linear regression model.

### Stratified analysis

**FIGURE 4:**
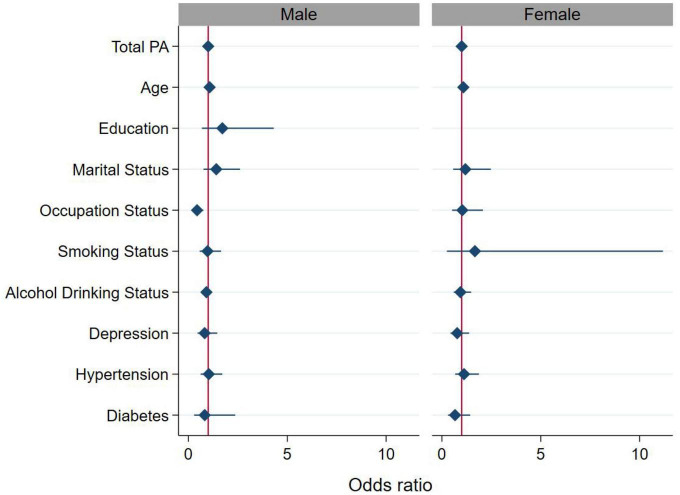
Association between total physical activity (PA) (continuous variable) and MCI stratified by gender. For the analysis, we adjusted for age, education level (≤12 years or >12 years), occupation status (farmers or others), marital status (married or others), smoking status (yes or no), alcohol drinking status (yes or no), depression, hypertension, and diabetes. The diamond and solid lines represent the estimated OR and its 95% confidence interval, respectively.

Table 5 and Figure 4 showed the relationship between total PA and MCI stratified by gender, and the interaction between gender and total PA on MCI. The results of the stratified analysis showed that the higher total PA level after adjusting for covariates, the lower their risk of MCI in men (*P* = 0.041), even women (*P* = 0.040). The results of the interaction analysis indicated that gender does not play a modifying effect in the relationship between MCI and PA level before and after covariates adjustment (*P*_int_ > 0.05).

**TABLE 5:** Stratified analysis by gender of the association between total PA and MCI.

	Case/control	OR (95%CI)^a^	*P* ^a^	OR (95%CI)^b^	*P* ^b^
Whole population	411/236	0.9998 (0.9998–0.9999)	0.001	0.9999 (0.9998–0.9999)	0.001
Male	159/140	0.9999 (0.9998–0.9999)	0.008	0.9999 (0.9998–1.0000)	0.041
Female	252/96	0.9998 (0.9998–0.9999)	0.001	0.9999 (0.9998–0.9999)	0.040
*P*_int_			0.158		0.741

^a^Crude results.

^b^Results adjust for age, education level (≤12 years or >12 years), occupation status (farmers or others), marital status (married or others), smoking status (yes or no), alcohol drinking status (yes or no), depression, hypertension, and diabetes.

## Discussion

Based on the data of the elderly health survey in 17 villages in Jimo District, Qingdao City, Shandong Province, this study explored the association between PA and cognitive function in the elderly. In the present study, PA was a risk factor for visual space, attention, language, abstract ability and delayed recall, and PA was a protective factor for MCI. Our study identified that PA was positively correlated with total cognitive function scores. There was a saturation effect between total PA and total cognitive function scores, and the saturation point was 6546 MET × min/wk. In the multiplicative interaction model, we found that there is no significant interaction between gender and PA.

The results of the association between PA group and cognitive function showed that PA was positively correlated with visual space, attention, language, abstract ability, delayed recall and total cognitive function scores, and PA is a protective factor for MCI. These findings are consistent with previous research. In randomized controlled trials, participation in exercise programs consistently improved cognitive performance regardless of baseline cognitive status (Brown et al., 2009; Barnes et al., 2013; Morris et al., 2017). A recent meta-analysis showed that PA interventions were effective in improving cognitive function (attention, executive function, memory and working memory, and overall cognition) in adults over 50 years of age (Northey et al., 2018).

The present study found a non-linear association between PA and cognitive function with saturation effect and saturation point. Previous studies have explored the relationship between PA and cognitive function, but the conclusions are controversial (Xu et al., 2017; Dupré et al., 2020; Petermann-Rocha et al., 2021; Iso-Markku et al., 2022). Some studies have found a non-linear association between PA and cognitive function, which is consistent with our findings (Dupré et al., 2020; Petermann-Rocha et al., 2021; Iso-Markku et al., 2022). A few studies have shown that there is no non-linear association between PA and cognitive function (Iso-Markku et al., 2022). A previous meta-analysis of the dose-response relationship have found that cognitive function has a linear relationship with PA in the observation range, and becomes non-linear when the range is expanded (Xu et al., 2017). However, few studies have investigated the specific threshold effect and saturation effect of PA on cognitive function. Our study not only found a non-linear relationship between PA and cognitive function, but also found a specific saturation effect and saturation point of 6546 MET × min/wk. This implies that PA levels are positively associated with cognitive function below the saturation point in older adults over 60 years of age, but when beyond the saturation point, the protective level of PA on cognitive function may not be additional.

It is worth noting that in our study only 21.2% of survey participants did not meet the minimum amount of PA recommended by WHO. A previous study reported that the proportion of inactive adults aged 60 or over in Southeast Asia is about 30% (Hallal et al., 2012). The proportion of people who did not meet PA standards in our study was lower than that in other studies. One possible reason was that the majority of our study population were farmers who engaged in more physical activities (Trost et al., 2002).

The possible mechanisms of how PA reduces cognitive decline are as follows. PA promotes cerebral blood circulation and cerebral blood flow redistribution, enhances antioxidant effect by increasing enzyme and pro-inflammatory cytokine activity (Lange-Asschenfeldt and Kojda, 2008). Additionally, PA can promote nerve regeneration and synaptic genesis by increasing vascular endothelial growth factor, brain-derived neurotrophic factor, nerve growth factor expression, and reducing the aggregation of Aβ plaques, thereby improving brain structure and neural circuits involved in cognition (Pereira et al., 2007; Gomez-Pinilla et al., 2011; Liu et al., 2011). Moreover, PA improves learning, memory, and cognitive function by reducing tau protein aggregation and increasing gray and white matter volume in the hippocampal and temporal cortical regions of the brain (Colcombe et al., 2006).

The risk factors for MCI involving biology, sociology and other fields, affect each other to jointly determine the risk of morbidity. Our study showed that gender had no moderating effect on the relationship between MCI and total PA. The mechanism is not well understood, possibly due to our small sample size not enough to elucidate this moderating effect. In addition, some studies have pointed out that the atypical clinical presentation of male MCI patients may lead to the occurrence of missed diagnosis (Liesinger et al., 2018).

There are also some limitations of this study that need to be addressed. First, the definition of MCI relatively simple, the method of defining MCI in this article is only used for screening MCI, not as a standard for diagnosing MCI. In the future, more research is needed to validate using strict diagnosing standards for MCI. Second, this study measured the amount of PA using IPAQ, which required participants to self-report and recall their PA in the previous week, which may led to recall bias. Some studies suggest that IPAQ may overestimate total PA. Apolipoprotein E4 (APOE4) genotype status was usually seen as an important factor for dementia. However, our study did not perform the APOE genotyping, so we failed to consider it as a covariate adjusted in regression models. Finally, this study is limited by the cross-sectional design, which excludes any conclusion about causation. A longitudinal study is warranted to confirm this association in the future.

## Conclusion

Together, our findings suggest that a higher PA level is associated with a lower risk of cognitive impairment. The current study confirmed the saturation effect between PA and cognitive function and determined an optimal level of PA to protect cognitive function. This finding will help update PA guidelines based on cognitive function in the elderly.

## Data availability statement

The raw data supporting the conclusions of this article will be made available by the authors, without undue reservation.

## Ethics statement

The studies involving human participants were reviewed and approved by Ethics Committee of Qingdao University Medical College. The patients/participants provided their written informed consent to participate in this study.

## Author contributions

XW, DZ, and SL conceived and designed the study. XW, JZ, CC, ZL, and SL investigated and collected the data. XW, JZ, and SL analyzed the data and wrote the draft. CC and ZL revised the manuscript. DZ and SL reviewed the manuscript and had primary responsibility for the final content. All authors have read and agreed to the published version of the manuscript.
